# The financial risk of real estate combined factor analysis with grey prediction in Liaoning Province

**DOI:** 10.1371/journal.pone.0301526

**Published:** 2024-04-30

**Authors:** Jian-Lin Yuan, Nan Jing

**Affiliations:** 1 School of Management, Liaoning University of Technology, Jinzhou, 121001, China; 2 Tangshan Letters and Calls Bureau of Hebei Province, Tangshan, 063000, China; Laboratoire Ville Mobilite Transport, FRANCE

## Abstract

The importance of real estate development has been widely accepted by all countries. Through early warning and avoidance of real estate financial risks, it can effectively promote the healthy and healthy development of the real estate industry, avoiding the impact of accidental factors, such as the COVID-19 pandemic, and promoting the overall economic development. Based on multiple regression analysis and grey prediction methods, this article constructs a real estate financial risk estimation model, and the real estate financial risk is estimated using the relevant data of Liaoning Province from 2001 to 2020. Analyzing the research results of financial risks in Liaoning Province, we can find that the real estate financial risks reached the peak in 2013, and then the real estate financial risks gradually showed a slow decline trend. In general, the financial risks in Liaoning Province are controllable. The study of financial risks in Liaoning Province will help to judge the development of the real estate industry and promote the continuous improvement of the overall economy. The article, through the study of real estate financial risks in Liaoning Province, can promote the development of regional real estate in Liaoning Province and promote the overall economic development of Liaoning Province, which has strong practical significance. The study of real estate financial risks, relevant risk research theories can be enriched, the identification of financial risks can be improved, and the study of real estate financial risks can be strengthened. The article uses a combination of multivariate statistics and grey fuzzy theory to complete the study of real estate financial risks. Therefore, through the exploration of multivariate statistics and grey fuzzy theory, its application value can be elevated.

## 1 Introduction

In 2023, real estate enterprises suffered serious financial risks, and the three famous real estate enterprises in China, namely Evergrande Group, Country Garden, and Rongchuang, faced difficulties in operation due to financial strain. In 2023, the debt amounts of Evergrande Group, Country Garden, and Rongchuang were RMB 2.4374 trillion, RMB 1.4349 trillion, and RMB 1.0038 trillion, respectively. The enormous financial risks faced by the real estate industry have become a hidden concern of industry development and a serious threat to the overall economic development. Avoiding financial risks in the real estate industry and promoting its healthy development has become a hot research topic at present. As the foundation and pillar industry in the development of national economy, the contribution of real estate to the whole national economy cannot be underestimated. However, in recent years, the sudden pandemic has seriously hindered the development of the real estate industry. This "black swan event" has brought a cold current to the real estate industry, which is known for its liquidity, and the pandemic has seriously affected its development, and then affected the overall economic development. As a capital-intensive industry, real estate is fixed and with investment characteristics, and needs a large amount of funds to support its development. The study of financial risks in the real estate industry will help promote its sustainable and healthy development, and escort the entire industry. Its significance is far-reaching. This article focuses on the perspective of regional economic development, with risk research as the entry point, highlighting the healthy and healthy development of the real estate industry, in order to drive overall economic growth.

The real estate industry has the characteristics of strong relevance and can effectively promote the prosperous development of multiple industries, such as building materials, home appliances, services, etc. Because of real estate impact on the development of the entire national economy, it is being given increasing amount of attention by scholars from different countries. Tang and Ge [1] analyzed the current financial risks in China’s real estate industry and believed that its mainly manifested in four aspects: (1) Commercial banks were the source of market and financial risks; (2) There was implicit risk in land reserve loans; (3) personal housing consumption credit had entered an internationally recognized period of high default risk; (4) There were management risks in the operation of commercial banks and other financial institutions. Corresponding countermeasures had been proposed for real estate financial risks. Li and Zhang [2] believed that the main problems of real estate financial risks in China were: (1) The scale of bank loans continued to expand; (2) The expansion scale of overseas capital markets continued to grow; (3) The debt ratio of real estate enterprises would continue to be high. And corresponding countermeasures were proposed based on their problems.

Through research on real estate financial risks, it is possible to effectively avoid potential real estate crises and promote their healthy development. Li [3] conducted early warning analysis on the financial risk status of Chongqing’s real estate based on the regression analysis model of support vector machine (SVM). Through the relationship between data indicators and output values, she analyzed the nonlinear relationship between the financial risk status of real estate and indicator variables, and predicted the financial risk status and trend of financial risk in each year. Zhang and Li [4] used the factor analysis method and the equal variance weight method to construct the real estate financial risk pressure index to complete the risk study of the real estate financial market in the western region of China, and concluded that the real estate financial risk in the western region of China is constantly increasing, and used ARIMA to predict the real estate financial risk in the western region of China, and put forward corresponding risk avoidance measures. Deng [5] used the ridge regression method to measure the real estate foam based on the real estate data of 31 provinces and autonomous regions in China from 2005 to 2019. The results showed that with the increase of the housing, the risk of the foam weakened, while the housing price income ratio increased, the debt of real estate enterprises increased, and the foam increased. Xiang and Cao [6] calculated the comprehensive early warning value of Chongqing’s real estate based on the efficiency coefficient method, took the real estate data of Chongqing from 2000 to 2020, so as to make a certain judgment on the real estate risk in Chongqing. Guo et al. [7] used the dynamic volatility spillover index method of generalized variance decomposition to measure the volatility spillover risk of China’s nine real estate finance sub-industries from 2011 to 2020, and concluded that the volatility spillover risk of China’s real estate finance continued to rise from 2019, which was consistent with the cyclical characteristics of monetary policy. Tang and Mao [8] used the spatial semi-parametric nonlinear Durbin model to study the path dependence and spatial spillover effects of China’s real estate risk, local government debt risk and their superposition mechanism. It is concluded that the real estate and local government debt risks had an adverse effect on the financial system; the superposition of real estate and local government debt risks also has a negative spatial spillover effect on the financial system.

By analyzing risk factors, risk trends can be controlled and potential risks can be predicted. Huang et al. [9] studied the return rate of the Credit Default Swap (CDS) index during the Federal Open Market Committee (FOMC) cycle in the United States. The results indicated that in even weeks of the Federal Open Market Committee cycle, the CDS return rate were significantly higher than in odd weeks. The biweekly pattern of the CDS market could be seen as a reflection of the stock market, with its trading strategy generating an annual excess return of 8.8%. This high return model was related to the schedule of the Federal Reserve’s internal board of directors’ biweekly meetings to address macroeconomic uncertainty. Through further evidence analysis, the Federal Reserve influenced the CDS market through unexpected information signals and monetary policy, thereby reducing risk premiums. Wu et al. [10] studied the predictability of stock return cross-sectional uncertainty (CSU). Studies showed that CSU had significant differences to predict monthly stock returns within and outside the sample, its returns was between 11.89% and 6.34%, respectively, higher than popular predictive factors. The use of a bi-variate combination of CSU and one of the alternative predictors produced an annual sample prediction return rate of up to 18.08%. CSU had brought significant economic benefits to general investors, with annual utility returns exceeding 400 basis points. The intensification of economic globalization had led to active cooperation among stock markets in various countries. Chen et al. [11] analyzed the risks faced by securities markets in the context of global cooperation through time series analysis.

Clarifying the financial risk characteristics of real estate can control the development trend of real estate and thus grasp the development of real estate. Zhou and Sun [12] used the principal component analysis method to complete the impact of monetary policy on the real estate full-financing risk through SVAR and threshold model. The results showed that the adjustment of monetary policy would have an impact on the real estate full-financing risk, and its impact on the real estate full-financing risk was asymmetric at different house price levels. Ju et al. [13] used the spatial measurement method to complete the impact of housing vacancy rate on real estate financial risk based on the panel data of 30 provinces from 2014 to 2015. The results showed that China’s real estate financial risk had certain spatial relevance, and the housing vacancy rate had a positive impact on real estate financial risk. Su Zheny et al. [14] used the Fama French three factor (FF3) model to conduct a real estate return risk analysis, and found that the change in return rate of S-REITs was 68.7%. In the long run, market factors had less explanatory power than scale and value factors; The positive long-term multiplier of the scale factor indicated that small S-REIT companies had higher returns and higher risks, while the negative multiplier of the value indicator indicates that the S-REIT portfolio tended to allocate growth REITs with lower book to market ratios.Xu et al. [15] used the real estate financial data of 31 provinces in China from 2006 to 2018 to study the risk of the real estate financial composite index. It was concluded that (1) the spatial correlation effect of China’s real estate financial risk was increasing, and presents the characteristics of multiple superposition and spatial spillover effect; (2) The risk in the eastern region was the highest, the risk in the central region was the middle, and the risk level in the northwest was higher; (3) The financial risk of real estate was formed by the high dependence of local financial resources on land financing. Lee et al. [16] used Portfolio Expected Loss (PEL α) and Portfolio Upper Loss (PUL α) to measure the financial risk of real estate and divide it into five quality levels in order to achieve better risk aversion effect. Larriva and Linneman [17] completed the real estate financial risk study by using the proportion of mortgage debt to the US GDP. The results showed that the future interest rate would remain at a low level and the real estate financial risk would remain at a low level. Ojo et al. [18] investigated the performance of commercial and residential real estate investment in the Ibadan real estate market. The general least squares (OLS) regression model was used to test the characteristics of inflation hedging, and Granger causality test was used to analyze the causal relationship between variables. The research showed that the Ibadan real estate market was still immature, and there was a one-way causal relationship between inflation and the return of the selected property type.

Through the analysis of the literature, we can see that the research on real estate finance is carried out from multiple levels and has promoted the healthy development of the real estate industry. However, research about real estate finance, there are still certain flaws in the methods, indicator selection, and the periodicity of real estate financial risks, which need to be further deepened. In addition, there is still limited literature on the regional financial risks in real estate, and continuous exploration is needed. Based on this, this article takes the regional real estate industry in Liaoning Province as the main body, and uses a combination of factor analysis and grey prediction method to study the financial risk issues in its development process, in order to solve the difficulties in the development of the regional real estate industry.

## 2 Methodology

### 2.1 Data

The data in this paper is mainly from the Statistical Yearbook of Liaoning in 2021 (https://tjj.ln.gov.cn/tjj/tjxx/xxcx/tjnj/index.shtml), which can be publicly available. And the calculation of financial risk indicators of real estate in Liaoning Province is completed according to relevant data.

When studying the real estate financial risk in Liaoning Province, the indicator system mainly draws on Sun [19] setting of real estate financial risk indicators to and the real estate financial risk indicator system is established in Liaoning Province, as shown in Table 1.

**Table 1:** Variables and their meanings.

name	meaning	unit
*x*_1_	Ration of growth rate of real estate development investment to GDP growth rate-RDITGD	%
*x*_2_	Ration of investment in real estate development to investment in fixed assets of the whole society-RIDFAS	%
*x*_3_	Growth rate of sales area of commercial housing-GRSACH	%
*x*_4_	Ration of construction area of commercial housing to floor space of buildings completed-RCCTFS	%
*x*_5_	Supply and sales ratio of real estate-SSRORE	%
*x*_6_	housing price-to-income ratio-HPTIR	%
*x*_7_	The ratio of the growth rate of real estate loans to the growth rate of all loans (real estate loans include personal home purchase mortgage loans)-RGRTGL	%

### 2.2 Model

#### 2.2.1 Principal component model

Let *x* is the m-dimensional random vector *X* = (*X*_1_,*X*_2_,⋯,*X*_*m*_)′, mean is *E*(*X*) = *μ* and covariance matrix is *D*(*X*.) = ∑.

*F* = (*F*_1_,*F*_2_,⋯,*F*_*m*_)′ (*m*<*p*) is an unobservable random vector, and *E*(*F*) = 0, *D*(*F*) = *I*_*m*_ (i.e., the variance of each component of F is 1 and not correlated with each other). Also set *ε* = (*ε*_1_,*ε*_2_,⋯,*ε*_*p*_)′ is not related to F, and

$$E(\varepsilon )=0,D(\varepsilon )=(\begin{array}{lll} \sigma_{1}^{2} & & \\ & \ddots & \\ & & \sigma_{D}^{2} \end{array})=diag(\sigma_{1}^{2},\sigma_{2}^{2},\cdots ,\sigma_{p}^{2})=\underline{\underline{def}}\;D(diagonal\;matrix)$$

Assuming that the random vector X satisfies the following model:

$$\left\{ \begin{array}{l} X_{1}-\mu_{1}=a_{11}F_{1}+a_{12}F_{2}+\cdots +a_{1m}F_{m}+\varepsilon_{1}\\ X_{2}-\mu_{2}=a_{21}F_{1}+a_{22}F_{2}+\cdots +a_{2m}F_{m}+\varepsilon_{2}\\ \;\cdots \;\cdots \;\cdots \\ X_{p}-\mu_{p}=a_{p1}F_{1}+a_{p2}F_{2}+\cdots +a_{pm}F_{m}+\varepsilon_{p} \end{array}\right.$$

The model is called factor model, represented by a matrix as:

$\underset{p\times 1}{X}-\underset{p\times 1}{\mu }=\underset{\left(p\times m\right)}{A}\underset{\left(m\times 1\right)}{F}+\underset{\left(p\times 1\right)}{\varepsilon }$ here *F* = (*F*_1_,*F*_2_,⋯,*F*_*m*_)′

Consider its linear transformation

$$\begin{array}{l} Z_{1}=a_{11}X_{1}+a_{12}+\cdots +a_{1m}X_{m}=a_{1}^{\prime }X\\ Z_{2}=a_{21}X_{1}+a_{22}+\cdots +a_{2m}X_{m}=a_{2}^{\prime }X\\ \;\vdots \;\vdots \;\vdots \;\vdots \;\vdots \\ Z_{m}=a_{m1}X_{1}+a_{m2}+\cdots +a_{mm}X_{m}=a_{m}^{\prime }X \end{array}$$

Here

$$\begin{array}{l} Var(Z_{i})=(a_{i}^{\prime }\sum a_{i})(i=1,2,\cdots ,m)\\ Cov(Z_{i},Z_{j})=(a_{i}^{\prime }\sum a_{j})(i,j=1,2,\cdots ,m) \end{array}$$

if $Z_{i}=a_{i}^{\prime }$ and $\begin{array}{l} a_{i}^{\prime }a_{i}=1\\ Var(Z)=MaxVar(a_{i}^{\prime }X) \end{array}$ so *Z*_*i*_ is the ith principal factor of X.

*F*_1_,*F*_2_,⋯,*F*_*m*_ is called common factor of X *ε* = (*ε*_1_,*ε*_2_,⋯,*ε*_*p*_)′, *ε*_1_,*ε*_2_,⋯,*ε*_*p*_ is called special factor of X; Generally, Common factor *A* = (*a*_*ij*_)_(*p*×*m*)_ has an effect on every component of X, but *ε*_*i*_ is only effect on *X*_*i*_. Moreover, there is no correlation between the special factors and between the special factors and all common factors.

The matrix *F*_1_,*F*_2_,⋯,*F*_*m*_ in the model is the coefficient matrix to be estimated, called the factor load matrix.

$A=(a_{ij})(i=1,2,\cdots ,p;j=1,2,\cdots ,m)$ is load on the jth factor of the i-th variable (referred to as the factor load).

#### 2.2.2 Grey prediction method

*2*.*2*.*2*.*1 Basic idea of grey prediction model*. Grey prediction is an important part of the grey system theory. Its prediction model extracts effective information from chaotic and scattered sample data, and makes it systematizeds and organizeds, its can discover the inherent patterns of changes in the research object system, in order to grasp the long-term characteristics of the research problem and achieve fuzzy quantitative prediction of future variables describing the problem. Common methods include GM (1, 1) model and grey Verhulst model. According to the functions and characteristics of the grey prediction model, the prediction model can be divided into sequence prediction, waveform prediction, interval prediction, disaster prediction and system prediction [20].

*2*.*2*.*2*.*2 GM (1*, *1) prediction model*. GM (1,1) model is the most widely used dynamic model in the grey prediction model. The mathematical model steps of GM (1, 1) model are [21]:

(1) Processing the original sequence data. the original number sequence is X^(O)^:

$$X^{\left(0\right)}=[X^{\left(0\right)}(1),X^{\left(0\right)}(2),\ldots ,X^{\left(0\right)}(n)]\;(k)\geq 0,k=1,2,\ldots ,n)$$

Performing a level test on this set of data and observing that is the data suitable for model construction? If the data level test fails, it is necessary to perform a "translation transformation" of the data and pass the level test, and then make predictions. The level inspection formula is as follows:

$$\lambda (k)=\frac{x^{\left(0\right)}(k-1)}{x^{\left(0\right)}(k)},k=2,3,4\cdots ,n$$

If the final λ is between (e^(−2/)n+1)).e^(2/n+1)), the next step of calculation can be carried out. accumulatingX^(O)^ and get the generated sequenceX^(1)^

$$x^{\left(1\right)}(k)=\sum_{i=1}^{k}x^{\left(0\right)}(i)(k=1,2,\ldots ,n)$$

$$X^{\left(1\right)}=[X^{\left(1\right)}(1),X^{\left(1\right)}(2),\ldots ,X^{\left(1\right)}(n)]\;(k)\geq 0,k=1,2,\ldots ,n)$$

(2) Generate sequence *Z*^(1)^ by performing nearest neighbor mean on*X*^(1)^

$$Z^{\left(1\right)}(k)=0.5\;X^{\left(1\right)}(k)+0.5\;X^{\left(1\right)}\;(k‐1)k=2,3,\ldots ,n$$

General the generated sequence *X*^(1)^ approximation follows exponential law, establish Grey differential equations:

$$X^{\left(0\right)}(k)+a\;Z^{\left(1\right)}(k)=\mu \;k=2,3,\ldots ,n$$

The corresponding whitening differential equation is:

$$\frac{dx(1)(k)}{dt}+a^{\left(1\right)}x(k)=u$$

Among them, a and u are undetermined constants, a is called the developmental grey number, and u is called the endogenous control grey number, which is a constant input to the system.

(3) Calculating the value of α, μ by least square method.

The prediction model is obtained that the least squares method is used:

$$U(\begin{array}{c} \hat{\alpha }\\ \hat{\mu } \end{array})={\left(B^{T}B\right)}^{-1}B^{T}Y$$

here Y = [*X*^(0)^ (1), *X*^(0)^ (2), …, *X*^(0)^ (n)]

$$B=[\begin{array}{l} -\frac{1}{2}[X^{\left(1\right)}(1)+X^{\left(1\right)}(2)\;1\\ \vdots \;\cdots \;\vdots \\ -\frac{1}{2}[X^{\left(1\right)}(n)+X^{\left(1\right)}(n-1)\;1 \end{array}]$$

Substitute the estimated value $\hat{\alpha },\hat{\mu }$ into the equation, *X*^(1)^ = [*X*^(1)^ (1), *X*^(1)^ (2), …, *X*^(1)^ (n)]

Obtain the time response equation by solving the first order ordinary differential equation. $x^{\left(\hat{0}\right)}(k+1)=(x^{\left(1\right)}-\frac{u}{a})e^{-ak}+\frac{u}{a}(k=1,2,\ldots ,n-1)$ (4) Perform relevant restoration processing on the prediction model to obtain the prediction sequence, so as to make relevant predictions of the problem:

$$x^{\left(\hat{0}\right)}(k+1)=x^{\left(\hat{1}\right)}(k+1)-x^{\left(\hat{1}\right)}(k)(k=1,2,\ldots ,n-1)$$

$$x^{\left(\hat{0}\right)}=[x^{\left(\hat{0}\right)}(1),x^{\left(\hat{0}\right)}(2),\cdots ,x^{\left(\hat{0}\right)}(n)]$$

(5) To test errors and ensure that the established grey model has high prediction accuracy and credibility, it is necessary to conduct error testing on the prediction results. The commonly testing methods include residual testing and post validation. This article adopts the residual test method; the process is as follows:

First step: Calculate residual sequence *e*^(0)^(*k*)

$$e^{\left(0\right)}(k)=x^{\left(0\right)}(k)-{\hat{x}}^{\left(0\right)}(k)$$

Second step: Calculate the relative error Δ_*k*_

$$\Delta_{k}=\frac{e(k)}{x^{\left(0\right)}(k)}\times 100\%$$

Third step: Calculate the average relative error $\bar{\Delta }$

$$\bar{\Delta }=\frac{1}{n}\sum_{k=1}^{n}\Delta_{i}$$

## 3 Results and discussion

### 3.1 Data processing

According to the Liaoning Statistical Yearbook in 2021 (publicly published in https://tjj.ln.gov.cn/tjj/tjxx/xxcx/tjnj/index.shtml), relevant data of the regional real estate financial risks in Liaoning Province were obtained and processed, as shown in Table 2.

**Table 2:** 2001–2020 Liaoning Province real estate financial risk index value.

Year	RDITGD *x*_1_	RIDFAS *x*_2_	GRSACH *x*_3_	RCCTFS *x*_4_	SSRORE *x*_5_	HPTIR *x*_6_	RGRTGL *x*_7_
2001	1.1314	0.2274	0.0780	2.1551	1.5808	10.5103	1.1872
2002	1.1083	0.2419	0.0845	2.3959	1.5533	9.7707	1.1183
2003	1.1390	0.2335	0.0997	2.4836	1.4273	9.5302	1.0045
2004	1.3331	0.2402	0.1115	2.7325	1.1441	9.0512	1.4512
2005	1.0057	0.2065	0.2061	2.8884	0.9530	8.7381	0.8329
2006	1.1299	0.2007	0.1562	2.9629	0.9608	8.3446	1.1187
2007	1.0927	0.2014	0.1999	3.7110	0.8171	7.9374	0.9534
2008	1.1240	0.2057	0.2243	3.8955	0.9352	7.0045	0.9872
2009	1.1513	0.2148	0.1130	4.6083	0.7500	6.7716	1.0039
2010	1.0818	0.2160	0.2133	5.9659	0.6613	6.2265	1.0642
2011	1.0752	0.2532	0.2042	5.4350	0.8384	5.4360	1.0136
2012	1.0876	0.2499	0.1179	5.9803	0.7293	5.0658	0.9648
2013	1.0795	0.2569	0.0953	6.7662	0.6621	4.7860	1.0863
2014	0.7812	0.2161	0.0519	6.2823	1.0681	4.7796	0.7972
2015	0.6703	0.1986	0.0015	9.0434	0.8267	5.0971	1.0483
2016	0.7586	0.3130	-0.2240	9.7310	0.7299	6.9395	1.8959
2017	1.0387	0.3429	0.0522	9.2913	0.6721	7.1861	1.2145
2018	1.1303	0.3754	0.0043	10.6499	0.5779	8.0680	0.8486
2019	1.0291	0.4073	0.0595	13.0873	0.4917	8.2026	1.1661
2020	1.0435	0.4172	0.0083	16.6591	0.3849	9.3494	0.8385
Mean value μ	1.0496	0.2609	0.0929	6.3362	0.8882	6.9142	1.0798
standard deviation δ	0.1477	0.0687	0.1006	3.8700	0.3209	1.6377	0.2399

Data source: Liaoning Statistical Yearbook in 2021.

### 3.2 Principal component analysis of real estate financial risk in Liaoning Province

According to the relevant data of real estate financial risk in Liaoning Province, the principal component method is used to complete the financial risk analysis of real estate in Liaoning Province. First of all, complete the standardization and normalization of the collected data related to real estate financial risk in Liaoning Province. Secondly, SAS software is used to complete the principal component analysis of real estate financial risk in Liaoning Province.

According to the results of the principal component analysis of real estate financial risk in Liaoning Province (Tables 3 and 4), the main factors affecting the real estate financial risk in Liaoning Province are selected. When selecting the main factor, it is generally completed according to its cumulative contribution rate. The number of main causes is determined according to the cumulative contribution rate, which is generally more than 80%. Based on this, three main factors are selected as the main components of real estate financial risk in Liaoning Province.

**Table 3:** Analysis of principal components.

components	characteristic value	Difference	proportion	accumulate
**1**	3.0127	1.4158	0.4304	0.4304
**2**	1.597	0.1592	0.2281	0.6585
**3**	1.4377	0.7934	0.2054	0.8639
**4**	0.6443	0.4587	0.0920	0.9560
**5**	0.1857	0.0926	0.0265	0.9825
**6**	0.0931	0.0637	0.0133	0.9958
**7**	0.0295		0.0042	1.0000

**Table 4:** Extracted principal component factor.

components	characteristic value	Difference	proportion	accumulate
**1**	3.0127	1.4158	0.4304	0.4304
**2**	1.597	0.1592	0.2281	0.6585
**3**	1.4377	0.7934	0.2054	0.8639

According to the real estate financial risk data of Liaoning Province, the calculation of its principal component analysis value is completed, as shown in Table 5.

**Table 5:** Principal component analysis of indicators.

name	unit
*x*_1_	0.8021
*x*_2_	0.9533
*x*_3_	0.9138
*x*_4_	0.9680
*x*_5_	0.8373
*x*_6_	0.8560
*x*_7_	0.7169

At the same time, SAS software is used to obtain the factor scores of the main components of real estate financial risk in Liaoning Province, as shown in Table 6.

**Table 6:** Factor score.

	Factor1	Factor2	Factor3
*x*_1_	0.5401	0.2409	0.6726
*x*_2_	-0.7818	0.2875	0.5094
*x*_3_	0.7289	-0.4881	0.3798
*x*_4_	-0.9440	-0.0635	0.2699
*x*_5_	0.7446	0.4409	-0.2975
*x*_6_	0.2830	0.7600	0.4453
*x*_7_	-0.2300	0.6648	-0.4712

In order to obtain the principal component weight of real estate financial risk in Liaoning Province, the principal component index weight is calculated according to the following formula and the principal component matrix table, and the results are shown in Table 7.

$$A_{i}=\frac{\sum_{j=1}^{3}a_{ij}^{2}}{\sum_{i=1}^{7}\sum_{j=1}^{3}a_{ij}^{2}}$$

j is the number of main components, j = 1, 2, 3; I is the number of indicators, i = 1, 2, 3,…, 7.

**Table 7:** Weights of principal component indicators.

*x*_1_	*x*_2_	*x*_3_	*x*_4_	*x*_5_	*x*_6_	*x*_7_
0.1326	0.1576	0.1511	0.1601	0.1385	0.1415	0.1185

### 3.3 Financial risk analysis of real estate in Liaoning Province

**Fig 1:**
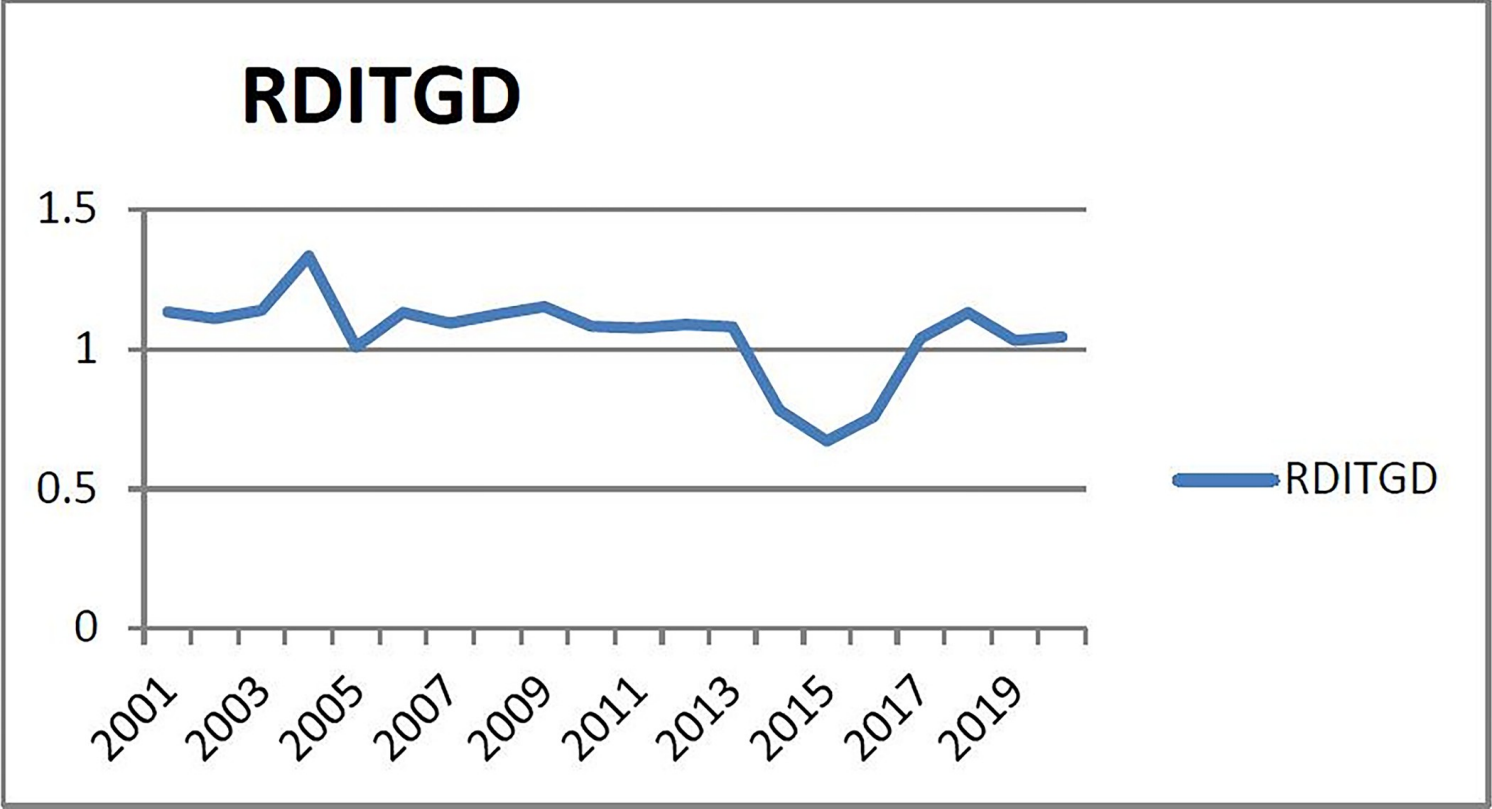
Relationship between real estate development investment growth rate and GDP growth rate in Liaoning Province from 2001 to 2020.

**Fig 2:**
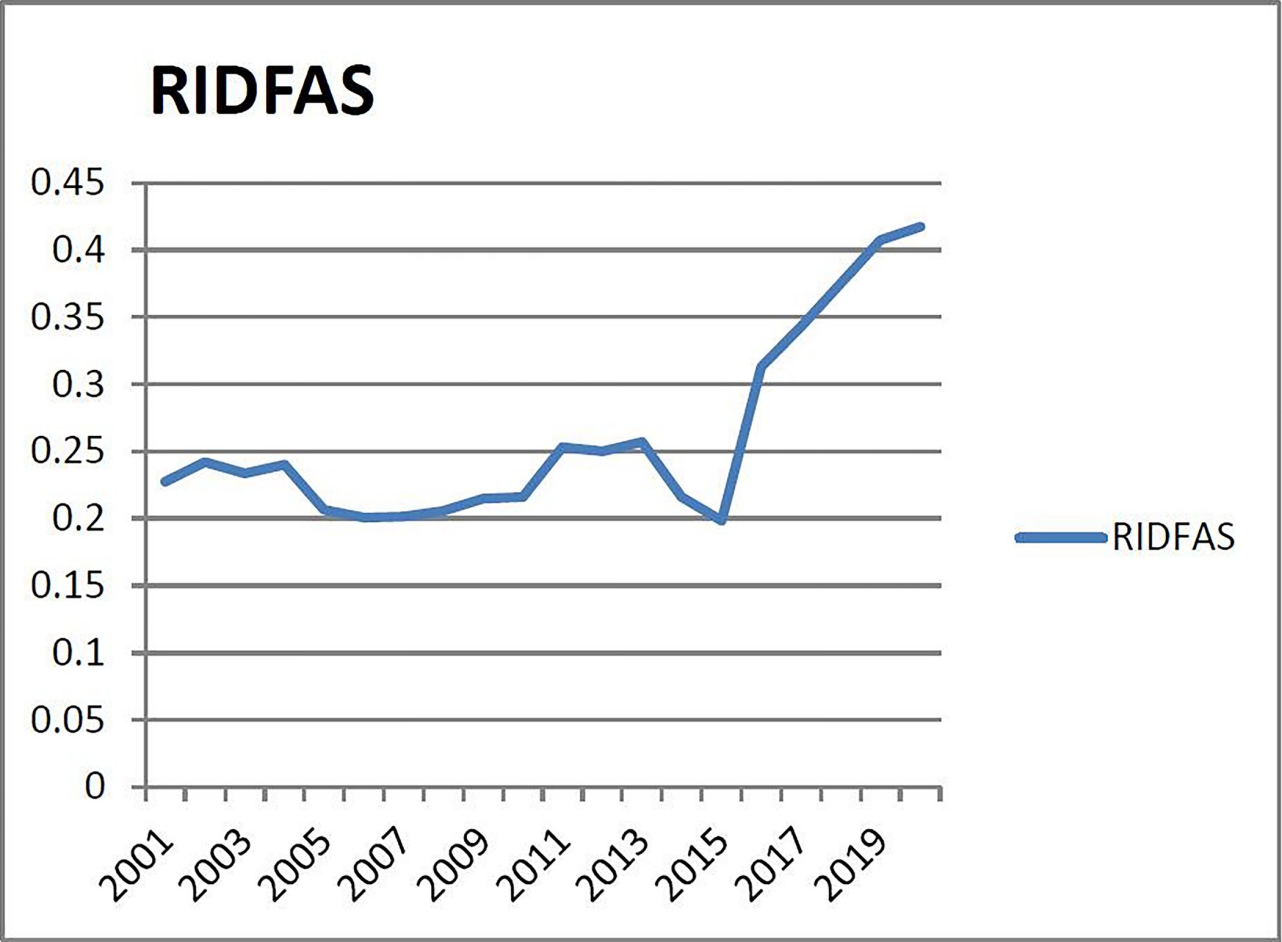
The ratio between real estate development investment and fixed asset investment of the whole society in Liaoning Province from 2001 to 2020.

**Fig 3:**
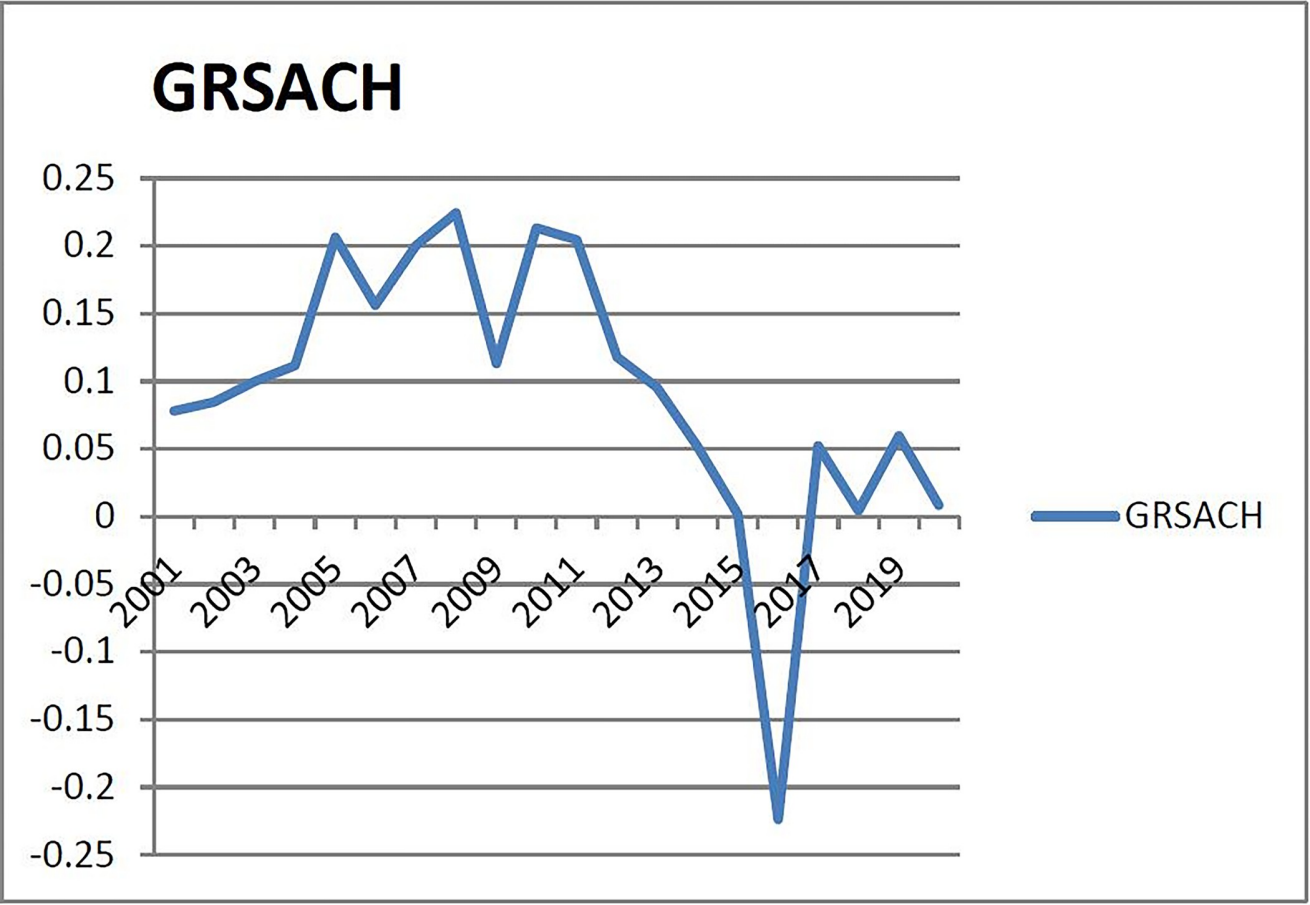
Growth rate of commodity sales area in Liaoning Province from 2001 to 2020.

**Fig 4:**
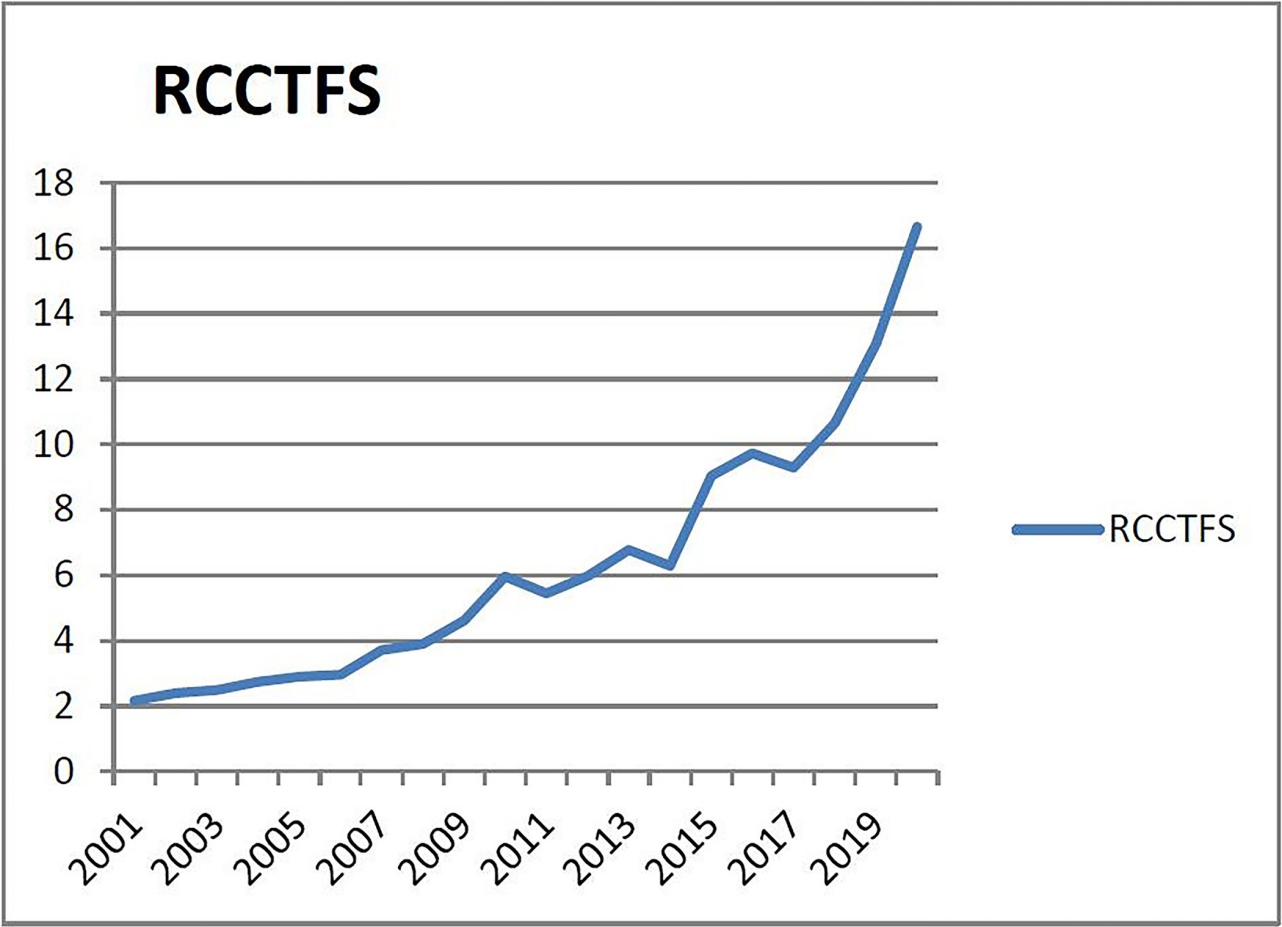
Relationship between the construction area of commercial housing and the completed area of commercial housing in Liaoning Province from 2001 to 2020.

**Fig 5:**
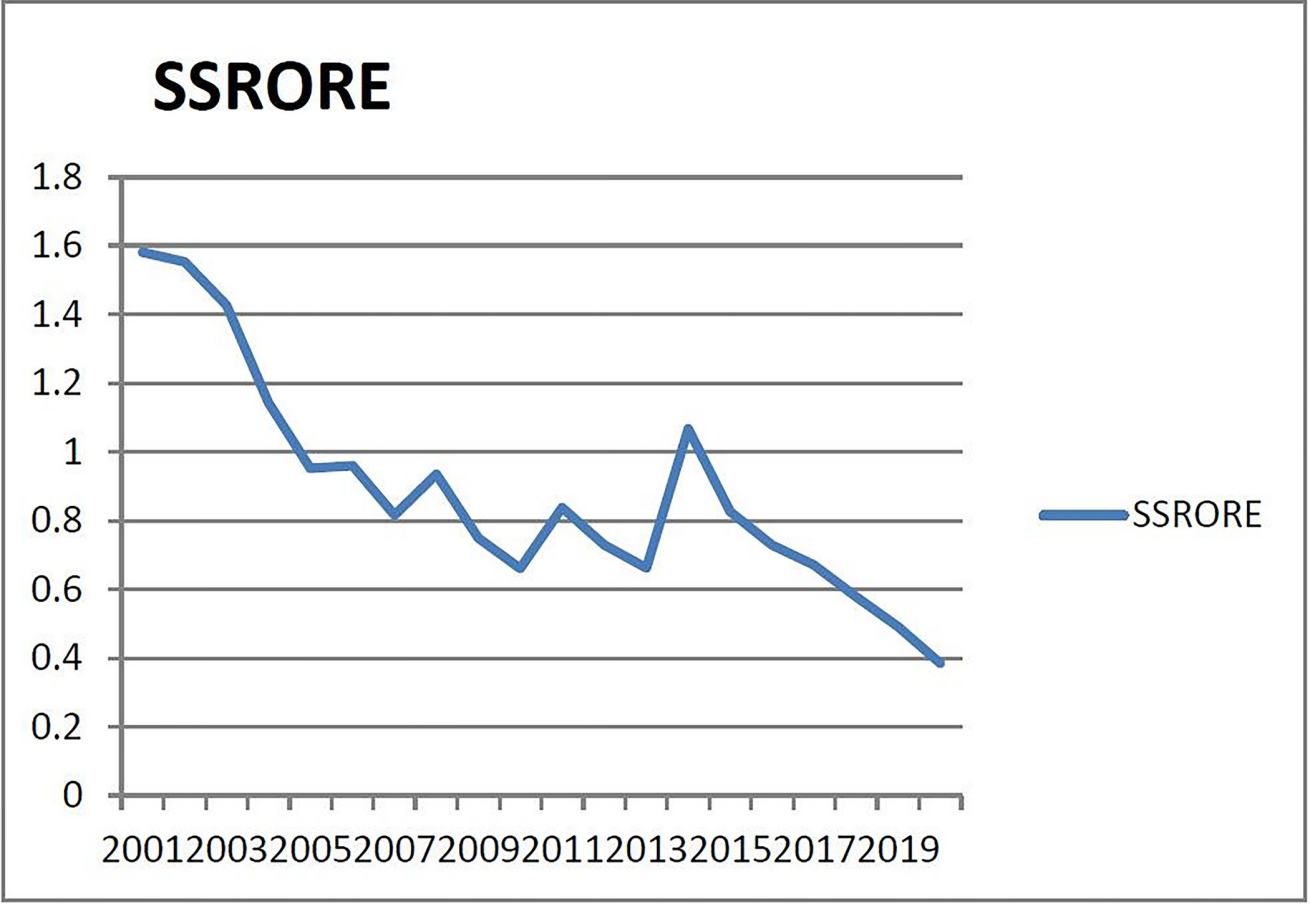
Relationship between supply and sales ratio of real estate in Liaoning Province from 2001 to 2020.

**Fig 6:**
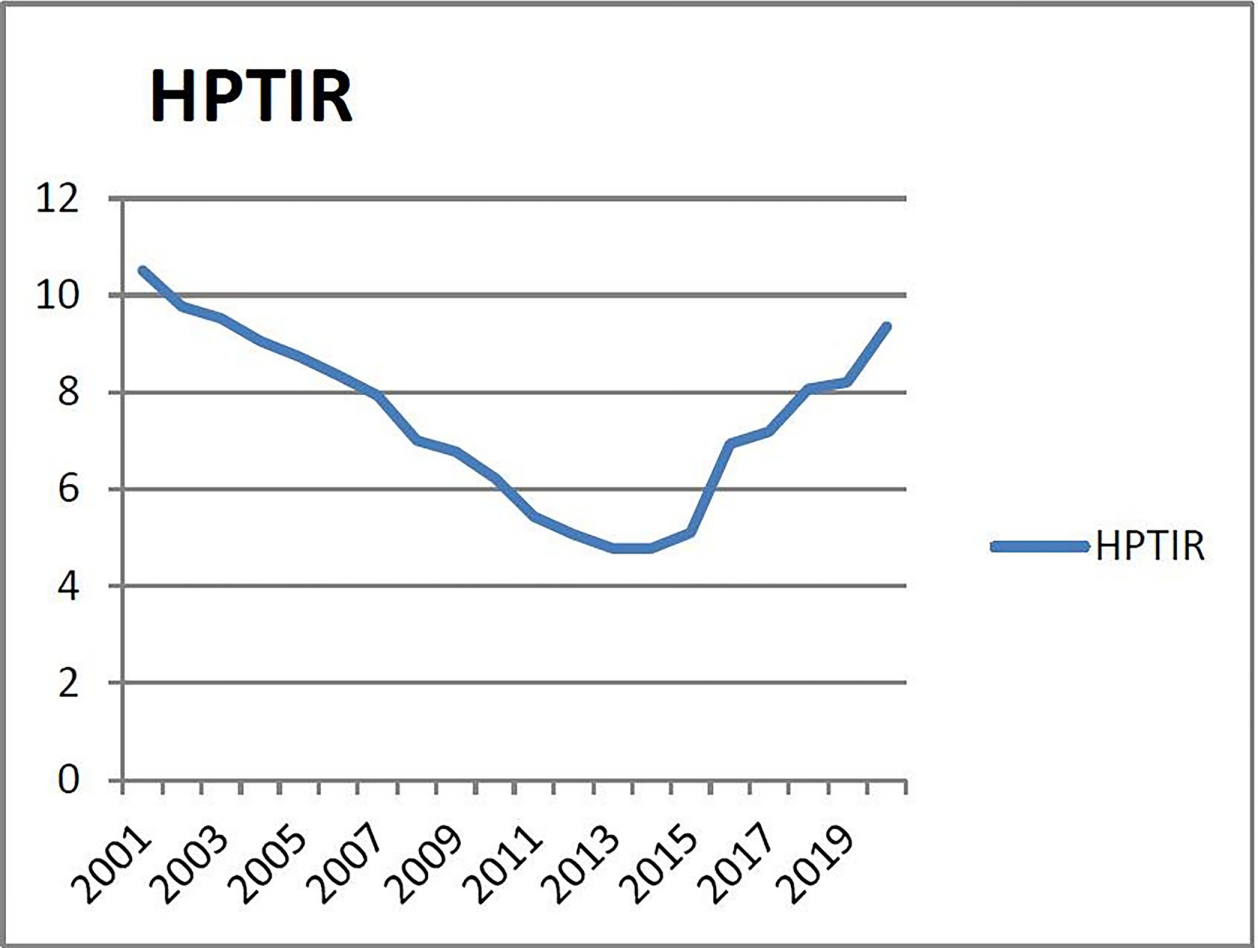
Relationship between house price and income ratio in Liaoning Province from 2001 to 2020.

**Fig 7:**
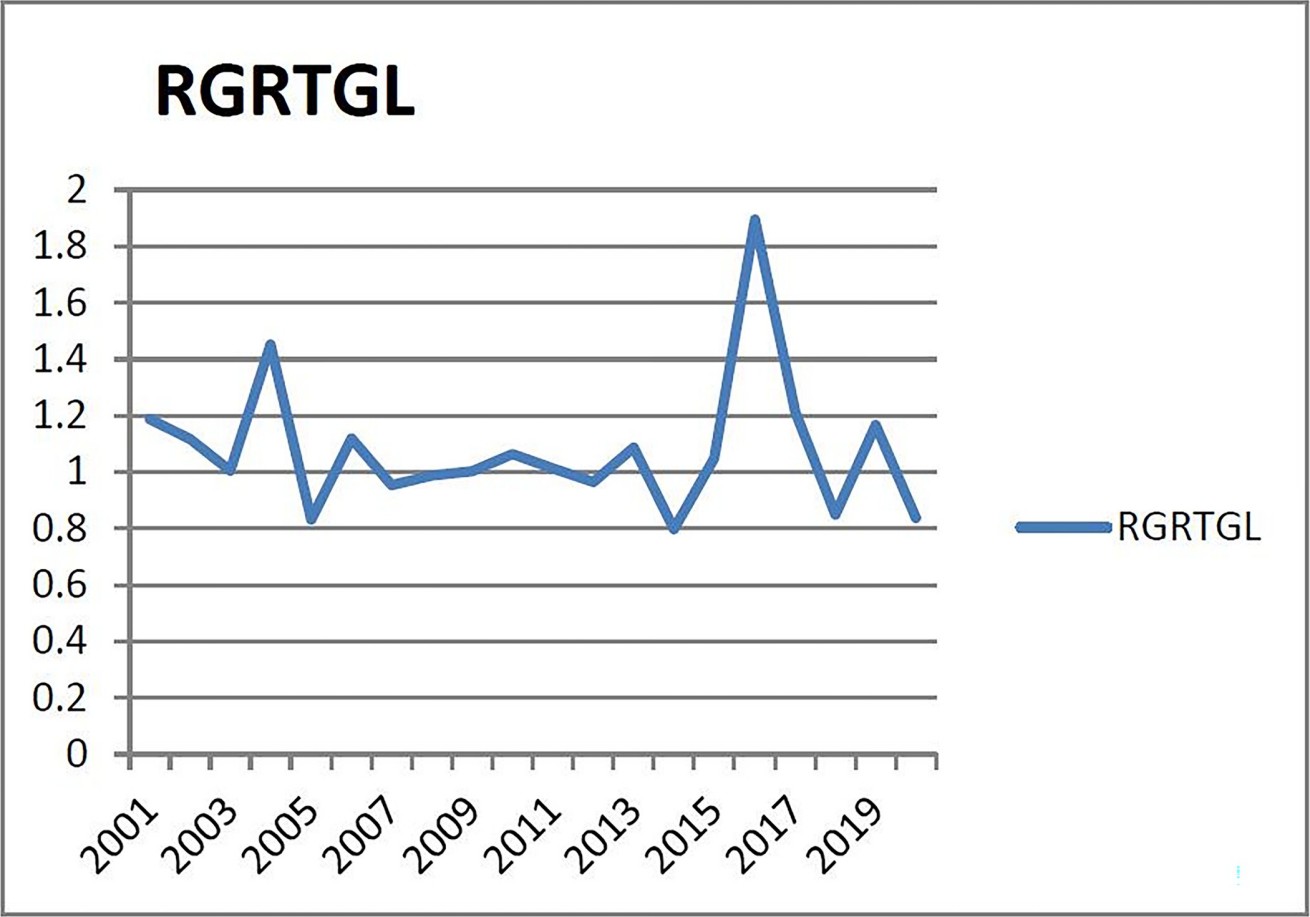
Relationship between real estate loan growth rate and total loan growth rate in Liaoning Province from 2001 to 2020.

(1) Checking the warning range of warning indicators of the first group (X_1_: the ratio of the growth rate of real estate development investment to the current GDP growth rate). From Fig 1, it can be seen that the ratio of real estate development investment growth rate to GDP growth rate in Liaoning Province was basically stable from 2001 to 2020. The X_1_ ratio shows three peaks in 2004 and 2020, three peaks are 2001, 2018, and 2020, respectively, the growth rate of real estate investment was a relatively high level during these three years; Between 2008 and 2015, there were three troughs, it is 2005, 2015, and 2019, the growth rate of real estate investment was at a relatively low level during these three years; and the high data of X_1_ index in each year was less than 2/3 of the year. Therefore, the warning interval is confirmed according to the economic situation, that is, the "half principle", and the alarm threshold interval is generally considered to be half of the year, No alarm in half of year (according to the Sun [22], the corresponding probability of no alarm zone in 50% of the year is 0.68 as the critical point of the normal interval), and the corresponding supercooled, slightly cooled, normal, slightly hot and overheated intervals are (-∞, μ-1.36σ], (μ- 1.36σ,μ- 0.68σ], (μ- 0.68σ,μ+ 0.68σ), (μ+ 0.68σ,μ+ 1.36σ), [μ+1.36σ,+∞). Therefore, the corresponding alarm limits of subcooling, micro-cooling, normal, micro-heat and overheating are (- ∞, 0.8487), (0.8487, 0.9491], (0.9491, 1.1500), (1.1500, 1.2504), (1.2504,+∞).

(2) Checking the warning range of warning indicators of the second group (X_2_: the ratio of real estate development investment to the total social fixed asset investment). As can be seen from Fig 2, the ratio of real estate development investment to social fixed asset investment in Liaoning Province increases steadily from 2001 to 2015, with an average of 12.56%, basically stable between 10% and 15%, lower than the proportion of real estate investment in developed countries accounting of social fixed assets investment for 20%~25%, indicating that the ratio of real estate investment and fixed asset investment in Liaoning Province is relatively coordinated. Therefore, based on the majority principle of overall preference, however, this indicator is at a low level in 2015, indicating that the real estate investment in 2015 was weak compared with the fixed assets investment of the whole society, 2/3 years are regarded as the non-alarm zone (according to the Sun [22], the corresponding probability of 2/3 years is 0.97 as the critical point of the normal zone), and the corresponding warning interval of subcooling, micro-cooling, normal, micro-heat and overheating are (-∞, μ- 1.94σ),(μ- 1.94σ,μ-0.97σ], (μ-0.97σ,μ+0.97σ),[μ+0.97σ,μ+ 1.94σ),[μ+ 1.94σ,+∞). Therefore, the corresponding alarm limits of subcooling, micro-cooling, normal, micro-heat and overheating are (-∞, 0.1276), (0.1276, 0.1943), (0.1943, 0.3275), (0.3275, 0.3942), (0.3942,+∞).

(3) Checking the warning interval of warning indicators of the third group (X_3_: growth rate of sales area of commercial housing) Fig 3 shows that the growth rate of sales area of commercial housing in Liaoning Province fluctuated greatly from 2001 to 2015, with an average annual growth rate of 16.89%, in 2016, the growth rate of commercial housing sales area was at a historical low point, due to the wing tail effect formed by the decline in real estate investment growth, of which the high data of each year is less than 2/3 years. Therefore, determine the warning interval according to the half principle to take the warning threshold, that is, considering that half of the year has a warning, there is no alarm in half of the year (according to the normal distribution probability table, the corresponding probability of no alarm zone in 50% of the year is 0.68 as the critical point of the normal interval). The corresponding subcooling, micro-cooling, normal, micro-heat and superheat ranges are (-∞, μ- 1.36σ), (μ- 1.36σ, μ-0.68σ), (μ- 0.68σ, μ+ 0.68σ), (μ+0.68σ, μ+ 1.36σ), (μ+1.36σ, +∞). Therefore, the corresponding alarm limits of subcooling, micro-cooling, normal, micro-heat and overheating are (-∞, -0.0453), (-0.0453, 0.0245), (0.0245, 0.1613), (0.1613, 0.2011), (0.2022, +∞).

(4) Checking the warning interval of warning indicators of the fourth group (X_4_: the ratio of the construction area of commercial housing to the multiple of the completed area of housing). From Fig 4, it can be seen that the ratio of the construction area of commercial housing to the completed area of housing in Liaoning Province has been steadily increasing from 2001 to 2015. In 2016, this indicator was at a historical low due to the lagging effect of slow real estate development in the previous year. The ratio of the construction area to the completed area of housing in recent 2/3 years is higher than the normal value (3 ± 0.5 times). Therefore, a few principles are adopted, that is, no alarm in 1/3 years and no alarm in 2/3 years. The probability of no alarm in 1/3 years is 43%, and the warning interval are (- ∞, μ- 0.86σ), (μ- 0.86σ, μ- 0.43σ), (μ- 0.43σ, μ+ 0.43σ), [μ+ 0.43σ, μ+ 0.86σ], (μ+0.86σ, +∞). The corresponding alarm limits of subcooling, micro-cooling, normal, micro-heat and overheating are (-∞, 3.008), (3.008, 4.6721), (4.6721, 7.9772), (7.9772, 9.6644), (9.6644, +∞).

(5) Checking the warning range of warning indicators of the fifth group (X_5_ real estate supply and sales ratio). Fig 5 shows that the supply and sales ratio of real estate in Liaoning Province is generally low from 2001 to 2015, and the supply and sales ratio of real estate in 2015 has remained above 50%, indicating that the real estate market in Liaoning Province has been in an active state for more than 10 years. There is a certain gap between the supply and sales of commercial housing in the market, there is a certain difference between the supply of real estate and the actual demand, and the supply of real estate is far from meeting the growth of demand, and the single indicator judgment is generally hot. Therefore, according to the principle of majority preference, 1/3 year is regarded as the non-pre-alarm area (according to the normal distribution probability table, the corresponding probability of 1/3 year is 0.43 as the critical point of the normal area), and the warning interval are (-∞, μ- 0.86σ), (μ-0.86σ, μ-0.43σ), (μ-0.43σ, μ+ 0.43σ), (μ+ 0.43σ, μ+0.86σ), (μ+ 0.86σ, +∞). Correspondingly, the numerical warning range of supercooling, micro-cooling, normal, micro-heat and overheating is (- ∞, 0.6123), (0.6123, 0.7502), (0.7502, 1.0262), (1.0262, 1.1641), (1.1641, +∞).

(6) Checking the warning range of warning indicator of the sixth group (X_6_: house price income ratio). From 2001 to 2015, the ratio of house price to income in Liaoning Province maintained a straight upward trend. Excluding the abnormal value of 2.9074 in 2001, the average value was revised to 6.0844 and the standard deviation was revised to 0.4777. According to Andrew HRmer, an expert of the World Bank, the ideal ratio of house price to income is 4~6 times which is an empirical value. In 2015, this indicator was at a relatively low level, which directly confirms that the real estate development in 2015 was in a relatively low state. From Fig 6, it can be seen that the ratio of house price to income in more than 2/3 years is basically about 6 times, indicating that the rise of house price in Liaoning Province is still high relative to the level of household income. Therefore, according to the principle of majority preference, the 2/3 year is regarded as the non-alarm zone (according to the normal distribution probability table, the corresponding probability of the 2/3 year non-alarm zone is 0.97 as the critical point of the normal zone), and the corresponding warning interval of supercooling, micro-cooling, normal, micro-heat and overheating are (-∞, μ- 1.94σ), (μ- 1.94σ, μ- 0.97σ), (μ- 0.97σ, μ+ 0.97), (μ+0.97σ, μ+1.94σ), (μ+ 1.94σ, +∞). Therefore, the corresponding alarm limits of subcooling, micro-cooling, normal, micro-heat and overheating are (- ∞, 3.7371), (3.7371, 5.6257), (5.6257, 8.5028), (8.5028, 10.0914), (10.0914, +∞).

(7) Checking the warning interval of warning indicators of the seventh group (X_7_: the ratio of the growth rate of real estate loans to the growth rate of all loans) Fig 7 shows that the growth rate of real estate loans in Liaoning Province between 2006 and 2015 (which the data cannot be counted before 2006) is higher than all the growth rate of loans, indicating that the weight of the real estate industry in the new loans of financial institutions is relatively high. The first growth peak appeared in 2007, the second peak appeared in 2010, and the growth in other years was relatively flat. Therefore, the half principle is still adopted to determine the alarm range of real estate growth ratio between 2004 and 2016, that is to say, half of the year has alarm and half of the year has no alarm (according to the normal distribution probability table, the corresponding probability of 1/3 year without alarm zone is 0.43 as the critical point of the normal interval). The corresponding subcooling, micro-cooling, normal, micro-heat and superheat ranges are (- ∞, μ-0.86σ), (μ- 0.86σ, μ-0.43σ), (μ- 0.43σ, μ+ 0.43σ), (μ+ 0.43σ, μ+ 0.86σ), (μ+ 0.86σ, +∞). Therefore, the corresponding alarm limits of subcooling, micro-cooling, normal, micro-heat and overheating are (-∞, 0.9166), (0.9166, 0.9766), (0.9766, 1.1829), (1.1829, 1.2429), (1.2429, +∞).

Sorting out the seven groups of warning ranges of warning indicators determined above, the numerical warning ranges of warning indicators X_1_~X_7_ are obtained (see Table 8).

**Table 8:** Early warning interval of warning indicators of real estate industry in Liaoning Province.

Index	Supercooling	Slightly Cold	Normal	Micro Heat	Overheated
*x*_1_	*x*≤0.8487	0.8487< *x*≤0.9491	0.9491< *x*<1.1500	1.1500≤*x*<1.2504	*x*≥1.2504
*x*_2_	*x*≤0.1276	0.1276< *x*≤0.1943	0.1943< *x*<0.3275	0.3275≤*x*<0.3942	*x*≥0.3942
*x*_3_	*x*≤-0.0453	-0.0453< *x*≤0.0245	0.0245< *x*<0.1613	0.1613≤*x*<0.2011	*x*≥0.2011
*x*_4_	*x*≤3.008	3.008< *x*≤4.6721	4.6721< *x*<7.9772	7.9772≤*x*<9.6644	*x*≥9.6644
*x*_5_	*x*≤0.6123	0.6123< *x*≤0.7502	0.7502< *x*<1.0262	1.0262≤*x*<1.1641	*x*≥1.1641
*x*_6_	*x*≤3.7371	3.7371< *x*≤5.6257	5.6257< *x*<8.5028	8.5028≤*x*<10.0914	*x*≥10.0914
*x*_7_	*x*≤0.9166	0.9166< *x*≤0.9766	0.9766< *x*<1.1829	1.1829≤*x*<1.2429	*x*≥1.2429

### 3.4 Liaoning Province real estate financial risk alarm judgment

**Fig 8:**
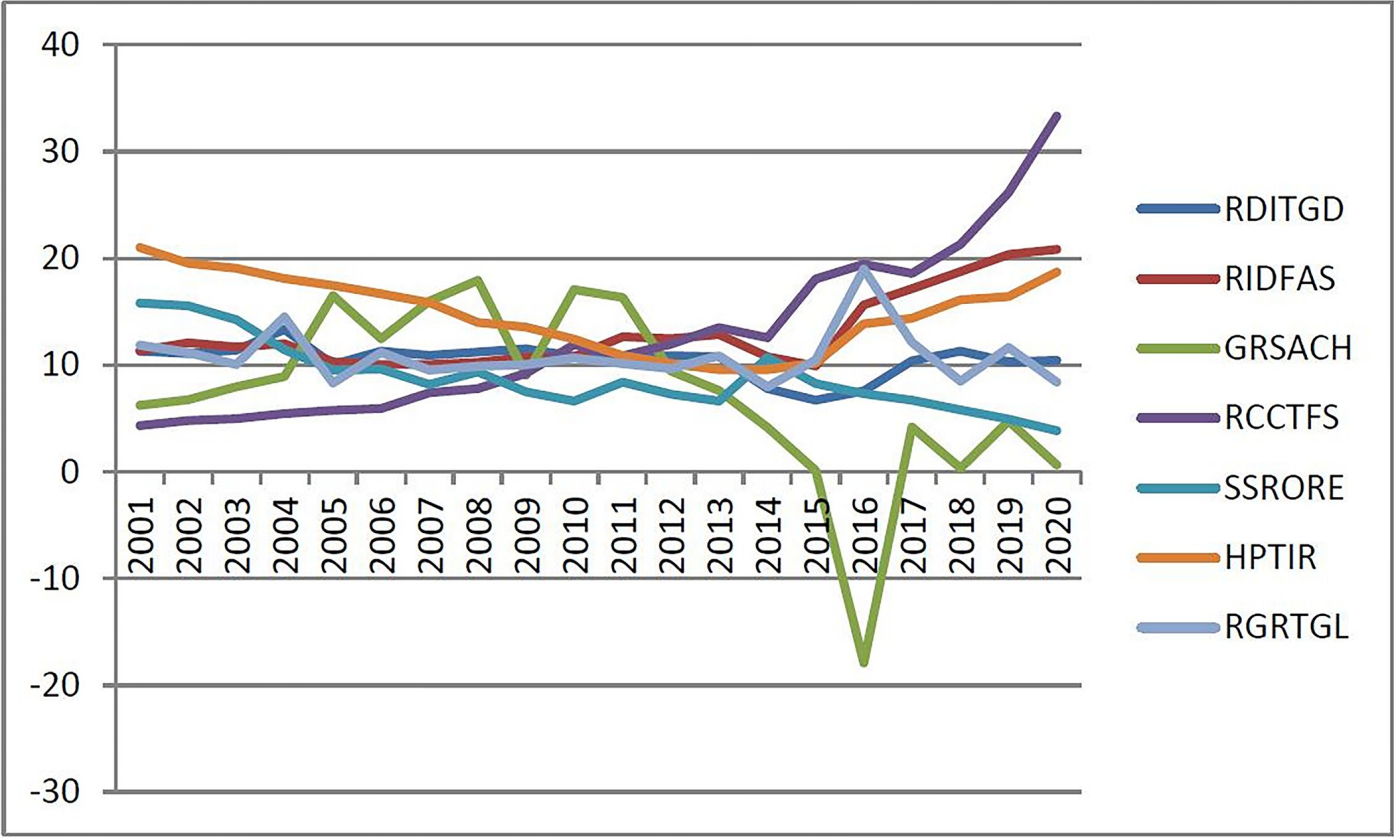
Distribution of real estate financial risks in Liaoning Province.

If judging the alarm situation of the real estate financial operation, all warning indicators should be comprehensively analyzed. With reference to domestic and foreign data, a group of signs is used to indicate the division of comprehensive warning interval that it is similar to traffic lights. The "red light, yellow light, green light, blue light and white light" can be used to intuitively reflect the "overheated, slightly hot, normal, slightly cold and excessively cold" state of the real estate financial market, and corresponding scores (5 points, 4 points, 3 points, 2 points and 1 point) are given respectively, then compare the warning indicators of Liaoning Province from 2001 to 2015 with the numerical warning interval in Table 7, and calculate the heat score of the real estate financial market operation in each year (see Table 8).Second according to the weight coefficients of the warning indicators X_1_~X_7_ calculated by the principal component analysis method, the weighted sum of the real estate market operation heat scores in each year is carried out, and finally the weighted score of the real estate financial market operation heat of Liaoning Province in each year from 2001 to 2015 is obtained. Referring to the real estate market early warning experience in Chongqing, Wuhan, Hubei and other places in China, and combining the real estate development in Liaoning Province in recent years, the interval ratio of the real estate financial market risk early warning was set at four critical points: 86%, 72%, 48% and 34% (domestic scholars also set the inter-area threshold value of early warning at 80%, 70%, 50% and 30%), and finally calculated the total score ratio of the real estate financial market operation heat for each year from 2001 to 2015, According to the critical value of domestic real estate early warning interval, the financial risk warning judgment of Liaoning Province from 2001 to 2020 is finally calculated (see Tables 9 and 10, Fig 8).

**Table 9:** Annual alarm value of real estate financial industry indicators.

year	RDITGD *x*_1_	RIDFAS *x*_2_	GRSACH *x*_3_	RCCTFS *x*_4_	SSRORE *x*_5_	HPTIR *x*_6_	RGRTGL *x*_7_
2001	Micro Heat	normal	normal	Supercooling	overheated	overheated	overheated
2002	Micro Heat	normal	normal	Supercooling	overheated	Micro Heat	Micro Heat
2003	Micro Heat	normal	normal	Supercooling	overheated	Micro Heat	normal
2004	overheated	normal	normal	Supercooling	Micro Heat	Micro Heat	overheated
2005	normal	normal	overheated	Supercooling	normal	Micro Heat	Supercooling
2006	Micro Heat	normal	normal	Supercooling	normal	Micro Heat	Micro Heat
2007	normal	normal	Micro Heat	slightly cold	normal	normal	slightly cold
2008	Micro Heat	normal	overheated	slightly cold	normal	normal	normal
2009	Micro Heat	normal	normal	slightly cold	slightly cold	normal	normal
2010	normal	normal	overheated	normal	slightly cold	normal	normal
2011	normal	normal	overheated	normal	normal	slightly cold	normal
2012	normal	normal	normal	normal	slightly cold	slightly cold	slightly cold
2013	normal	normal	normal	normal	slightly cold	slightly cold	normal
2014	Supercooling	normal	normal	normal	Micro Heat	slightly cold	Supercooling
2015	Supercooling	normal	Supercooling	Micro Heat	normal	slightly cold	normal
2016	Supercooling	normal	Supercooling	overheated	slightly cold	normal	overheated
2017	normal	Micro Heat	normal	Micro Heat	slightly cold	normal	Micro Heat
2018	normal	Micro Heat	normal	overheated	Supercooling	normal	Supercooling
2019	normal	overheated	normal	overheated	Supercooling	normal	normal
2020	normal	overheated	Supercooling	overheated	Supercooling	Micro Heat	Supercooling

**Table 10:** Real estate financial risk alarm judgment in Liaoning Province from 2001 to 2020.

year	Weighted score	Highest score	Total score proportion	Lamp type	Risk judgment
2001	3.6091	5	72.18%	green light | Yellow light	normal→Micro Heat
2002	3.3491	5	66.98%	green light	normal
2003	3.2306	5	64.61%	green light	normal
2004	3.4617	5	69.23%	green light	normal
2005	2.8862	5	57.72%	green light|blue light	normal→slightly cold
2006	3.0721	5	61.44%	green light| blue light	normal→slightly cold
2007	2.8722	5	57.44%	green light| blue light	normal→slightly cold
2008	3.2744	5	65.49%	green light	normal
2009	2.8337	5	56.67%	green light| blue light	normal→slightly cold
2010	3.1634	5	63.27%	green light	normal
2011	3.1604	5	63.21%	green light	normal
2012	2.6012	5	52.02%	green light| blue light	normal→slightly cold
2013	2.7197	5	54.39%	green light| blue light	normal→slightly cold
2014	2.4945	5	49.89%	green light	normal
2015	2.4509	5	49.02%	blue ligh |blue light	normal→slightly cold
2016	2.8510	5	57.02%	green ligh|t blue light	normal→slightly cold
2017	3.2974	5	65.95%	green light	normal
2018	2.9635	5	59.27%	green light| blue light	normal→slightly cold
2019	3.3581	5	67.16%	green light	normal
2020	2.9604	5	59.21%	green light| blue light	normal→slightly cold

### 3.5 Financial risk prediction of real estate in Liaoning Province

In this paper, the GM (1, 1) model is established based on the data in Table 8. With the help of the grey prediction model GM (1, 1), the comprehensive alarm value of real estate finance in Liaoning Province in 2016 and 2017 is predicted. With the help of the professional software of the grey system, the following gray indicators are obtained: the development gray level a = -0.023 internal control gray level μ = 2.4605.

According to the forcasting model formula ${\hat{x}}^{\left(1\right)}(k+1)=(x^{0}(1)-\frac{\mu }{a})e^{ak}+\frac{\mu }{a}$ The predicted comprehensive alarm values of the real estate financial market risk in Liaoning Province in 2016 and 2017 are calculated to be 3.5329 and 3.6152 respectively, and the accuracy of the model is tested:

(1) Difference post test

Mean square deviation of original sequence

$$S_{1}=\frac{\sum_{i=1}^{n}{\left[x^{0}(i)-\bar{x^{0}}\right]}^{2}}{n-1}$$

Mean square deviation of residual sequence

$$S_{2}=\frac{\sum_{i=1}^{n}{\left[\Delta (i)-\bar{\Delta }\right]}^{2}}{n-1}$$

The standard deviation of the original sequence is S_1_ = 0.595. The standard deviation of the residual sequence is S_2_ = 0.0606. The posterior error can be calculated C = S_2_/ S_1_ = 0.0606/0.595 = 0.1019, C≤0.35

(2) Small error probability test

S_0_ = 0.6745*0.595 = 0.40133

$e_{i}=|\Delta (i)-\bar{\Delta }|=$ {0.015,0.188,0.14,0.419,0.351,0.178,0.251,0.071,0.299,0.207,0.257,0.218,0.109,0.279,0.098,0.223}

Small error probability can be obtained p = 0.93

The test shows that the accuracy is level 1, GM (1, 1) model can be used.

Set up four empirical critical points, namely, 86%, 72%, 48% and 34% of the early-warning interval ratio of the real estate financial market risk, and calculate the proportion of the comprehensive alarm value of the real estate financial market risk predicted by the above grey model in 2019 and 2020, 3.5329 and 3.6152, respectively, to obtain the total score ratio of 70.65% and 72.3% in 2019 and 2020, thus predicting that the alarm status of the real estate financial market in Liaoning Province in 2019 is "normal" But it is the upper boundary of "normal tendency to slight heat". Referring to the actual operation of the real estate financial market in Liaoning Province in the first quarter of 2016, the real estate development investment and commercial housing construction area in the province increased by 7% and 7.5% year-on-year, respectively year on year in the first quarter, and the market investment tends to be rational and stable. However, the sales area of commercial housing still showed a relatively high growth rate. In the first quarter, the sales area of commercial housing increased by 23.5% year on year, of which the sales area of existing residential housing increased by 41.2% year on year, which is related to the series of policies that the country has continuously issued to relax the regulation and control of the real estate financial market since this year. At present, the development of the real estate financial market in Liaoning Province is basically consistent with the results of this forecast. The forecast result of the real estate financial market in the whole province in 2020 is "slight heat", but it is lower boundary of the "slight heat" region.

In summary, the real estate financial market in Liaoning Province developed well from 2001 to 2020, and it is in a relatively benign development trend. However, it is only a part of the national real estate financial market, due to the inertia of the national real estate development environment, the impact of the national phased macro-control factors and the impact of external effects such as the financial crisis, especially in the current weak economic recovery, the real estate market lurks certain industrial and financial risks, which need to be paid attention to.

### 3.6 Avoidance of financial risk of real estate

In response to the above warning judgments, regional financial institutions in Liaoning Province, based on the needs and considerations of supporting local economic development, effectively maintaining financial stability, and preventing regional financial risks induced by real estate risks, we can adopt different color levels (red, yellow, blue, and white) of risk prompts for different warning situations, for example, in the red level, measures should be taken such as tightening monetary policy, controlling the issuance of real estate loans, and limiting consumer purchases; When the warning level is yellow, financial restriction measures can be taken in a limited extent to avoid the continuous escalation of real estate financial risks; When the warning level is blue, and certain positive policies can be adopted to promote the development of the real estate industry; When the warning level is white, proactive policies can be adopted to increase the inclusive policies of the real estate industry and enhance its development potential. According to research on real estate warning, it can provide guidance and suggestions to relevant departments to ultimately ensure the safety of regional real estate finance, promote the healthy development of the real estate market.

## 4 Conclusion and limitation

### 4.1 Conclusion

At present, research on real estate financial risk warning is still in the process of continuous improvement, and related research methods and their promoting effect on economy need to strengthened.

1. This article constructs a risk warning and monitoring system for the real estate financial market in Liaoning Province, and uses principal component analysis and grey prediction GM (1,1) model to empirically study the real estate financial risks in Liaoning Province from 2001 to 2020. The conclusion of real estate financial risk situation is consistent with the actual situation.
2. From the overall trend of real estate development in Liaoning Province, the risk of development is relatively low, which can be attributed to two aspects: first, the stability of the overall economic development in Liaoning Province, ensuring that the volatility of its real estate industry is not high, and the impact of financial risk on the industry is limited; second, the industrial policy positioning of the real estate industry in Liaoning Province is clear, forming a healthy development of the industry, and the financial risks of the real estate is lower.
3. According to the research on real estate financial risks in Liaoning Province, the overall trend is currently developing well, but there is still a certain upward trend in risks in some years. The volatility of individual real estate should not be ignored due to the overall situation being positive. Efforts should be made to prevent financial risks and avoid potential impacts on regional economic development. However, due to limitations in the knowledge level of researchers and the sensitivity of government departments to the real estate market, which leads to difficulties in data collection, the research results have certain limitations, and the relevant warning methods still need further revision and improvement.

### 4.2 Limitations

The article provides a useful discussion on the financial risks of real estate in Liaoning Province, which provides some reference for regional economic development. However, there are also certain limitations in the research.

1. When conducting relevant research on the financial risk of real estate in Liaoning Province, the data obtained is the average data of the region, and the economic data indicators of individual regions are not marked, so the overall financial risk is relatively low, which does not necessarily represent the potential hidden risks in the development of the real estate industry in individual regions.
2. The methods of real estate finance risk research in Liaoning Province, is only certain studied in a certain scope, and detailed analysis of the problem has been made as much as possible. However, the research methods will be polished to accurately demonstrate the essence of the problem.
3. The analysis of real estate finance risk in Liaoning Province is only based on conclusions drawn from relevant data. In the analysis of the problem, there are certain differences due to different times and periods, which can be further discussed over time.
